# Scleroglucan as Structure Forming Agent of Low-Fat Yogurt: Effects on Functional Properties, Bacterial Activity and Sensory Profile

**DOI:** 10.3390/molecules30234581

**Published:** 2025-11-28

**Authors:** Marek Aljewicz, Marika Magdalena Bielecka, Aneta Dąbrowska, Małgorzata Anna Majcher, Łukasz Popławski

**Affiliations:** 1 Department of Dairy Science and Quality Management, Faculty of Food Science, University of Warmia and Mazury in Olsztyn, Oczapowskiego 7, 10719 Olsztyn, Polandaneta.dabrowska@uwm.edu.pl (A.D.); 2 Faculty of Food Science and Nutrition, Poznań University of Life Sciences, Wojska Polskiego 31, 60624 Poznan, Poland; 3 Department of Market and Consumption, The Faculty of Economic Sciences, University of Warmia and Mazury in Olsztyn, Plac Cieszynski 1, 10720 Olsztyn, Poland

**Keywords:** β-glucan, volatile compounds, fermentation, protein-polysaccharide interaction, hydrocolloid

## Abstract

Background: scleroglucan, an extracellular polysaccharide with gel-forming, thickening, and stabilizing properties, was used as a structure-forming agent in low-fat yogurt formulations. The aim of this study was to evaluate its influence on the fermentation process and the physicochemical, rheological, textural, microstructural, and sensory properties of the yogurts. Methods: control samples were formulated with the addition of skim milk powder (SMP), whereas experimental yogurts contained scleroglucan at concentrations of 0.25%, 0.5%, and 1.0% (*w*/*w*). The fermentation kinetics, acidity, color, syneresis, rheological behavior, texture profile, microstructure, and volatile compounds were analyzed during storage. Results: the results showed that scleroglucan slowed acidification and increased the apparent viscosity, yield stress, and firmness of yogurts, while completely eliminating syneresis. Scleroglucan also modified the volatile profile by decreasing acetaldehyde and increasing 2,3-pentanedione levels during storage. The survival of *Streptococcus thermophilus* and *Lactobacillus delbrueckii* subsp. *bulgaricus* was not affected. Conclusions: the yogurt containing 1.0% scleroglucan was rated highest in overall acceptability. These findings demonstrate that scleroglucan can serve as a natural, clean-label stabilizer and an alternative to skim milk powder in low-fat set-style yogurts.

## 1. Introduction

Yogurt is one of the most commonly consumed dairy products, whose appeal stems from both its high nutritional value and characteristic consistency. Changes in consumer preferences require modifications to production technologies and the development of products that, in addition to their nutritional value, also exhibit health-promoting properties. The development of low-fat, low-sugar, and high-protein yogurts plays a special role. Although these products meet consumer health expectations, their production can be associated with technological challenges, including syneresis and reduced sensory acceptability. During the fermentation process and storage of yogurts, intensive biochemical transformations occur, including the proteolysis of casein and whey proteins. These changes affect both the product’s consistency and its overall taste. The casein network remains the key element responsible for the structure of yogurt. The integrity of this network determines the gel consistency, and its microstructural properties depend on factors such as the type of milk and technological conditions. Syneresis, defined as the shrinkage of the protein matrix and the secretion of whey, is one of the most serious technological challenges in yogurt production. Its intensity depends on the microstructure of the gel. It is known that the use of polysaccharides can modify the susceptibility of the protein matrix to syneresis. Some additives intensify this process, whereas others stabilize the gel network and consequently reduce whey separation [1].

In dairy production, milk powder or milk protein concentrates are commonly used to stabilize the product and increase dry matter content and improve the protein network and rheological characteristics of yogurt. Polysaccharides such as gellan gum, xanthan gum, sodium alginate, and carrageenan are widely used to improve texture and reduce syneresis through interactions with milk proteins [2,3,4]. However, despite their effectiveness, these hydrocolloids are often perceived negatively by consumers due to their perceived artificial origin and labeling requirements, which drives interest in natural alternatives.

In recent years, there has been growing interest in the use of β-glucans as natural stabilizers in yogurt production. These polysaccharides, found in yeast, mushrooms, and grains, can improve the textural and rheological properties of yogurt. In addition to their technological effects, they also provide significant health benefits; they exhibit immunomodulatory, cholesterol-lowering and anti-cancer properties, which makes them attractive additives in the context of functional foods [5]. The addition of β-glucans to yogurt recipes not only increases viscosity, improves water retention, and reduces syneresis, but also enables the production of functional products that meet current market trends [6,7,8]. It is important to note that β-glucans are not a single, uniform class with identical behavior in dairy systems. Cereal β-glucans from oat and barley are linear, mixed-linkage (1→3)(1→4)-β-D-glucans that are highly water-soluble and viscosity-forming; their techno-functional effect strongly depends on molecular weight, extraction, and solubility, which, in set-type yogurts, can translate into shear-thinning, serum binding, and starter-culture interactions distinct from other β-glucans [9,10].

Despite their health benefits, cereal β-glucans present several technological limitations in low-fat dairy matrices. The beneficial effect of β-glucans in dairy products depends on the molecular weight of the polysaccharide, the type of additive used, and the fat content. In systems with low fat content and high β-glucan concentration (>0.5%), due to their interaction with milk proteins at their isoelectric point and their high thermodynamic incompatibility, they can cause severe gel shrinkage and whey separation [9]. Consequently, their application in reduced-fat and low-calorie yogurt formulations remains challenging.

In view of the above, scleroglucan, a non-ionic β-glucan produced by the fungus *Sclerotium rolfsii*, appears to be particularly interesting. Its chemical structure consists of a linear chain of β-(1→3)-D-glucopyranose units, every third of which is branched by a β-(1→6)-D-glucopyranose bond [11]. A characteristic feature of scleroglucan is the presence of a stable triple helix, which gives it high rigidity, resistance to hydrolysis, and stability in a wide range of temperatures and environmental conditions [12]. Moreover, even a small amount (>0.25%) significantly increases the viscosity of the system [9]. From the technological perspective of low-fat yogurt production, it is particularly important to find additives that provide the desired consistency and stability with limited fat content. In practice, this means that a small amount of scleroglucan can effectively strengthen the structure of yogurt, reducing syneresis and improving consistency, which is particularly important in low-fat products. Scleroglucan differs from both cereal β-glucans and curdlan in structural motif and mode of action in milk: the (1→3)-β-backbone with regular (1→6)-β-branches assembles into very stiff, salt- and acid-tolerant triple helices that remain cold-soluble and impart viscosity at low dosages without heat-triggered gelation; this combination supports protein-matrix reinforcement and serum immobilization during set-yogurt formation, while minimizing thermal processing constraints characteristic of curdlan [13]. Scleroglucan combines the desired structural stability of curdlan with the cold-water solubility of cereal β-glucans, making it a unique candidate for clean-label, low-fat yogurt formulations.

Polysaccharides used as food additives can affect the acidifying activity of starter cultures in various ways, which has a direct impact on the dynamics of fermentation and the synthesis of volatile compounds in dairy products. Their effect depends on their chemical structure, physicochemical properties, and concentration. Carrageenan and xanthan gum affect the proliferation of *Enterobacteriaceae* bacteria, which can also interfere with the activity of starters [14]. Oat β-glucan is also important—adding it in concentrations above 0.25% delays the fermentation process and reduces the number of starter bacteria during storage, leading to a slowdown in acidification [9]. The above studies also showed that the use of polysaccharides, such as oat β-glucan and curdlan, modifies the profile of volatile aromatic compounds in yogurt. The addition of oat β-glucan at a concentration above 0.25% results in decreased acetaldehyde concentration from approximately 8.5 µg/kg in the control samples to 5.0 µg/kg, which could weaken the characteristic aroma of yogurt. In contrast, curdlan promotes increased production of buttery–creamy compounds—the concentration of diacetyl in the control increased from 2.0 µg/kg to 3.1–3.4 µg/kg, and acetoin from 32 µg/kg to 45–50 µg/kg—enriching the sensory profile of the product. On the other hand, some polysaccharides have a stimulating effect. Pullulan used in concentrations of 1.5–2.0% significantly extends the survival of *Bifidobacterium animalis* subsp. *lactis* in yogurt, maintaining their numbers above therapeutic levels during storage [15]. Starch and its derivatives, such as maltodextrin or glucose-fructose syrup, accelerate fermentation and improve the survival of lactic acid bacteria during storage, while stabilizing the physicochemical properties of the product [16]. The use of β-glucans in yogurts not only improves the stability and technological quality of the product, but also improves its nutritional and health-promoting value. Previous research on β-glucans has focused mainly on oat, barley, and yeast fractions, while knowledge about scleroglucan remains limited. In particular, its influence on fermentation kinetics, starter-culture metabolism, and volatile compound profiles that shape yogurt aroma has not yet been comprehensively investigated. Furthermore, there is a lack of data on consumer acceptance and sensory performance of scleroglucan-enriched yogurts.

Therefore, the objective of this study was to investigate the effect of scleroglucan concentration (0.25–1.0%) on the acidification kinetics, physicochemical composition, rheological and textural parameters, microstructure, volatile profile, and sensory quality of low-fat set yogurts. The study also aimed to evaluate whether scleroglucan could serve as a natural structuring agent and a functional alternative to skim milk powder in yogurt manufacturing.

## 2. Results and Discussion

As shown in Table 1, the composition of the control and experimental yogurts differed in terms of dry matter. In the control samples, dry matter values ranged from 12.63% to 13.50% and were slightly lower than in the experimental yogurts, which ranged from 12.89% to 14.17%. As can be seen in Table 1, these levels were lower than the typical values reported for traditional commercial yogurts, which usually have a dry matter content of 14–16% [17]. Intentionally maintaining the dry matter content at a lower level made it possible to limit the stabilizing effect of natural milk components, such as casein, lactose, and fat, which in turn enabled a more precise assessment of the role of polysaccharides in shaping the structural properties of the product. The literature emphasizes that an increase in dry matter content improves the consistency and water retention of yogurt, but at levels exceeding 14%, milk proteins take over the dominant stabilizing function, which may weaken the visible effect of polysaccharide additives. In the analyses carried out, the protein content was similar in all samples, as a result of raw-material standardization, and ranged from 3.97 to 4.53%. These values correspond to the typical protein level in yogurts produced from skim milk. The fat content, in line with the use of skim milk, remained very low (0.05–0.07%), which was important for eliminating the influence of this component on the textural and sensory properties of the tested samples.

### 2.1. Acidity and Acidification Dynamics

**Figure 1:**
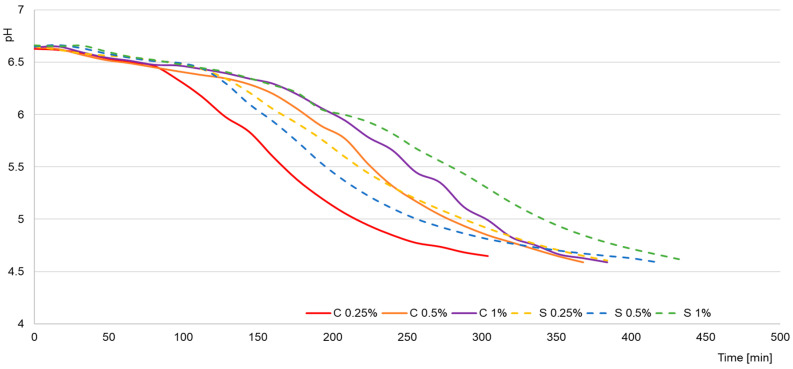
Acidification curves of milk. C—control samples; S—experimental samples containing scleroglucan. The numbers 0.25%, 0.5%, and 1% indicate the concentration of scleroglucan in the experimental yogurts and the corresponding level of skimmed milk powder in the control samples.

**Figure 2:**
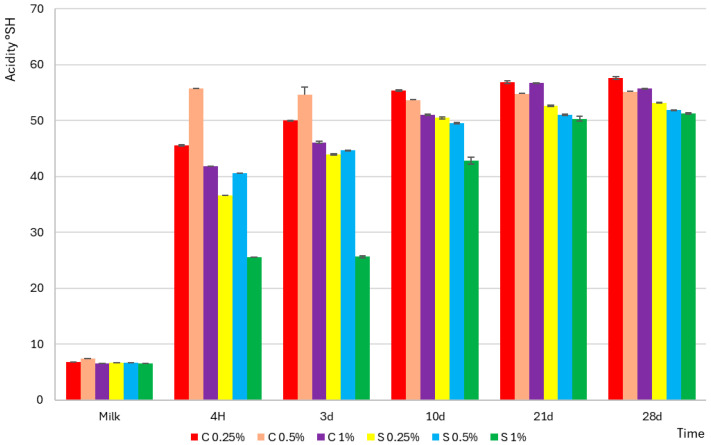
Potential acidity expressed in Soxhlet–Henkel degrees (°SH) of control (C) and experimental (S) yogurt samples containing scleroglucan. “Milk” refers to the acidity of the sample before inoculation with starter cultures, while “4 h” denotes the end of fermentation. Measurements were performed over 28 days of refrigerated storage. The values 0.25%, 0.5%, and 1% indicate the concentration of scleroglucan in the experimental yogurts and the corresponding level of skimmed milk powder in the control samples. Data are presented as means (n = 3), with error bars representing standard deviations (SD).

The early stages of fermentation were characterized by a more rapid acid production of *Streptococcus thermophilus*, while *Lactobacillus delbrueckii* subsp. *bulgaricus* initiated intensive acidification in the later phase, generating the greatest amount of lactic acid at the end of the process, as illustrated in Figure 1. Raw milk was characterized by low acidity (6.8–7.4 °SH) and high pH (6.63–6.75), and Figure 2 confirms that the addition of scleroglucan did not significantly affect the initial values (e.g., 0.25% C–6.63 °SH; pH 6.63; 0.25% S–6.72 °SH; pH 6.72). After fermentation, the faster acidification rate observed in the control yogurts is clearly visible in Figure 2, where the 0.25% C sample reached pH 4.6 after approximately 310 min, compared with 384 min for 0.25% S and 432 min for 1% S.

This was accompanied by an intense increase in the lactic acid concentration. In 0.25% C, it increased from 0.048 g/100 g to 0.316 g/100 g after 4 h and to 0.400 g/100 g on day 28, while in 0.25% S, the values were 0.046 g/100 g, 0.254 g/100 g, and 0.369 g/100 g, respectively. The observed relationships shown in Figure 2 indicate that the decrease in pH and increase in acidity were directly related to the accumulation of lactic acid, and the scleroglucan content determined both the rate and the extent [18]. The slower acidification observed in the samples containing scleroglucan can be attributed to limitations in substrate and metabolite diffusion caused by the increased viscosity and density of the protein–polysaccharide matrix. The triple-helical conformation of scleroglucan creates a thickened environment that reduces the mobility of lactose, amino acids and dissolved oxygen, which slows the glycolytic activity of starter cultures. Restricted oxygen transfer also modifies the redox balance, reducing the efficiency of NAD^+^ regeneration needed for sustained lactic acid synthesis. Such viscosity-driven constraints on fermentation kinetics were also demonstrated for matrices enriched with oat β-glucan or curdlan, where increased environmental density reduced the metabolic activity of *Streptococcus thermophilus* and *Lactobacillus delbrueckii* subsp. *bulgaricus* [19]. During storage (days 3–28), Figure 2 shows a milder increase in acidity and a slight decrease in pH. After 28 days, the control yogurts reached values of 57.6 °SH (0.25% C; pH 4.28), 54.7 °SH (0.5% C; pH 4.23), and 56.7 °SH (1% C; pH 4.22). Yogurts with scleroglucan showed lower final values: 53.2 °SH (0.25% S; pH 4.30), 51.8 °SH (0.5% S; pH 4.27), and 51.2 °SH (1% S; pH 4.22). These results were consistent with the lower lactic acid concentration in the experimental samples, confirming that scleroglucan reduced acid production during storage. Lower titratable acidity values corresponded directly to lower lactic acid levels, indicating that the presence of this polysaccharide limited the rate of acid synthesis, with the effect being most pronounced at the 1% level. From a technological and sensory point of view, this effect can be beneficial, as it helps to maintain a milder taste profile and greater stability of the yogurt matrix during storage.

### 2.2. Organic Acid Analysis

**Figure 3:**
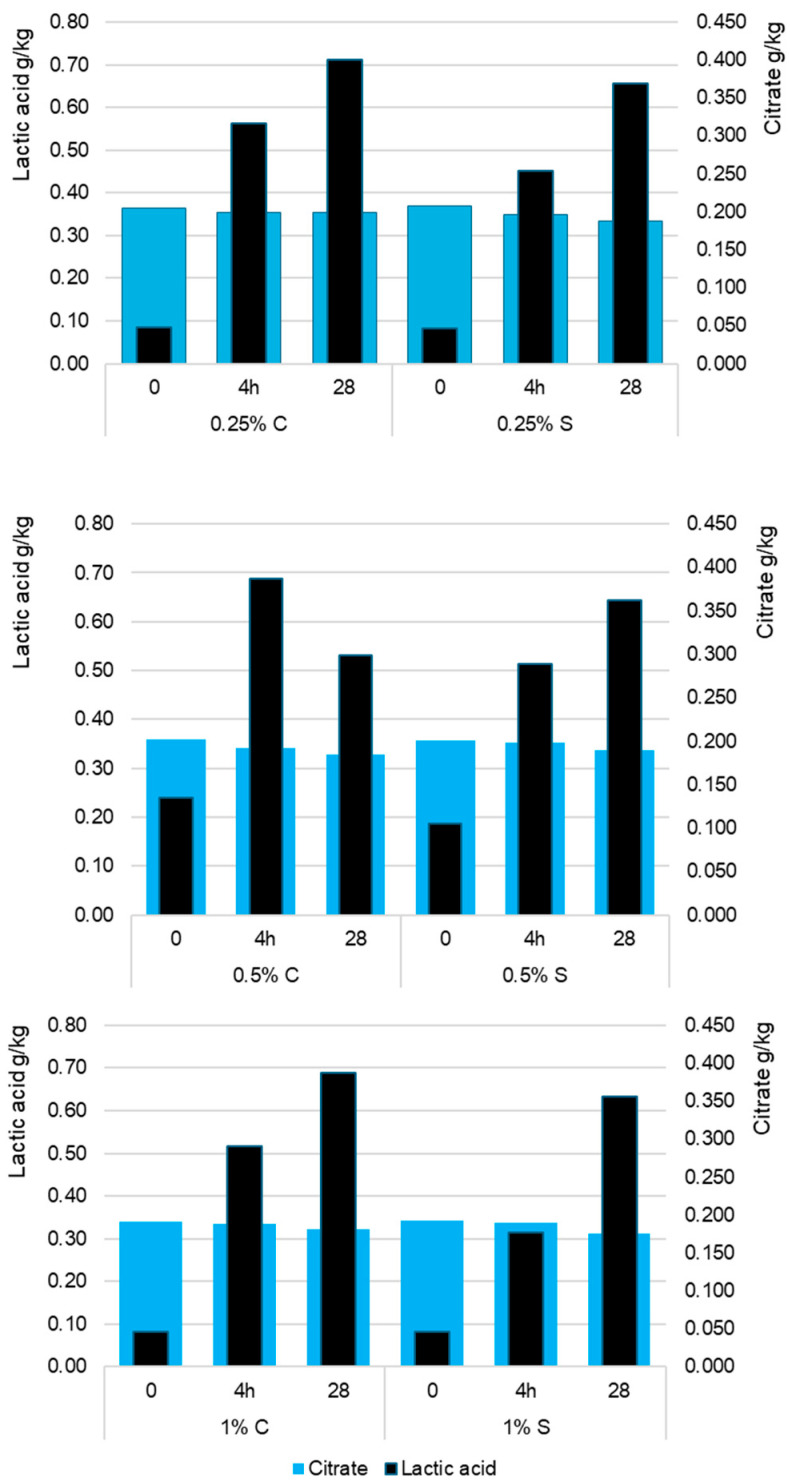
Average content of lactic acid and citrate (g/kg) in control (C) and experimental (S) yogurt samples measured after fermentation (4 h) and after 28 days of refrigerated storage. Time 0 refers to raw milk before inoculation with starter cultures. The values 0.25%, 0.5%, and 1% indicate the concentration of scleroglucan in the yogurt samples. Data are presented as means (n = 3). S denotes yogurts containing scleroglucan.

Statistical analyses showed that the citrate content in yogurts depended more on storage time (*p* < 0.001; η^2^p = 0.612) than on scleroglucan concentration (*p* < 0.01; η^2^p = 0.124), which is illustrated in Figure 3. The initial citrate level in raw milk (0.34–0.37 g/kg) remained stable after fermentation, with minor differences between 0.25% S (0.348 g/kg) and 0.25% C (0.354 g/kg) as shown in Figure 3. During storage, citrate gradually decreased, reaching 0.334–0.312 g/kg in the scleroglucan variants and 0.353 g/kg in the control. The lowest final values occurred at the 1% scleroglucan level. This effect likely resulted from limited substrate diffusion in the more viscous gel matrix and potential modifications in lactic acid bacteria metabolism. Because *Streptococcus thermophilus* and *Lactobacillus delbrueckii* subsp. *bulgaricus* do not metabolize citrate, the observed declines should be attributed to physicochemical rather than enzymatic processes. The increasing compactness of the protein–polysaccharide network led to partial immobilization of citrate ions in the gel structure and reduced their mobility in the aqueous phase, which lowered the measurable fraction during storage. This effect intensified with higher scleroglucan concentrations due to greater viscosity and reduced serum separation. These findings correspond with earlier reports indicating that citrate in fermented milk remains largely stable and that minor decreases result from physical redistribution rather than microbial degradation [19,20]. In the present study, the slight reduction in citrate content therefore reflects structural reorganization associated with scleroglucan-induced gel strengthening rather than changes in bacterial metabolism.

### 2.3. Sugar Metabolism (Lactose, Glucose, Galactose)

**Figure 4:**
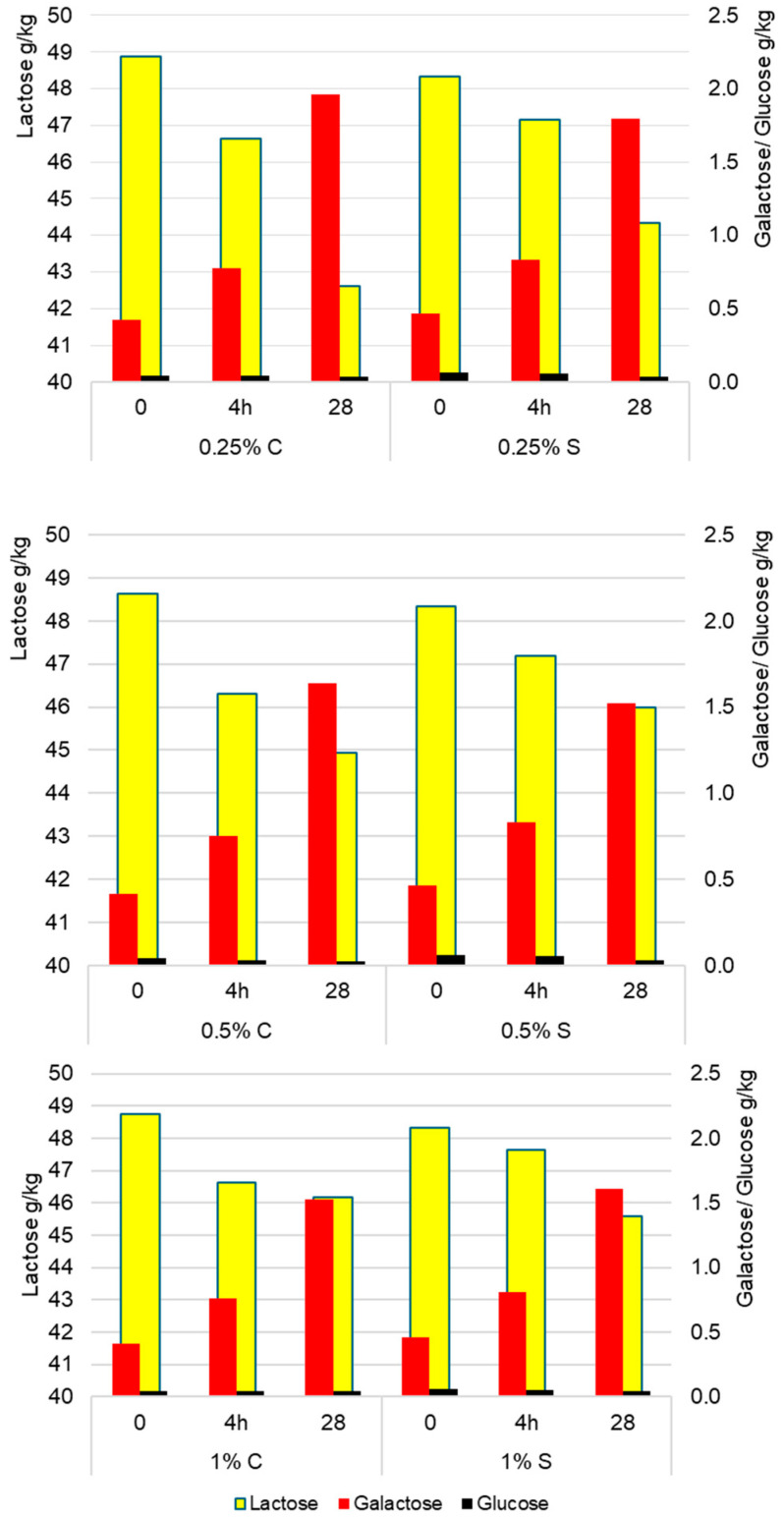
Average content of lactose, glucose, and galactose (g/kg) in control (C) and experimental (S) yogurt samples measured after fermentation (4 h) and after 28 days of refrigerated storage. Time 0 refers to raw milk before inoculation with starter culture.

Lactose metabolism depended significantly on both storage time (*p* < 0.001; η^2^p = 0.723) and the concentration of scleroglucan (*p* < 0.001; η^2^p = 0.410). The greatest changes occurred in the first 4 h of fermentation, when a rapid decrease in lactose concentration was accompanied by an increase in simple sugars, associated with enhanced β-galactosidase activity. In the later stages (4 h–28 days), the rate of change slowed down but remained statistically significant (*p* < 0.001). As can be observed in Figure 4, the highest rate of lactose conversion was found in yogurt with 0.25% scleroglucan, where after 28 days the lactose content decreased to 44.34 g/kg, and at the same time the highest accumulation of galactose (1.80 g/kg of product) and a minor reduction in glucose (0.03 g/kg). These results indicate that variants with a higher polysaccharide concentration were characterized by slower lactose hydrolysis, resulting in higher final lactose levels (45.99 g/kg for 0.5% S and 45.59 g/kg for 1% S) and lower galactose contents (1.52 g/kg and 1.61 g/kg, respectively) in Figure 4. The analysis of variance (ANOVA) showed a significant effect of the polysaccharide concentration on lactose content (*p* < 0.001). The differences between variants were +1.65 g/kg (0.25 vs. 0.5%), +1.25 g/kg (0.25 vs. 1.0%), and −0.40 g/kg (0.5 vs. 1.0%). These results indicate that higher doses of scleroglucan slowed lactose hydrolysis and monosaccharide accumulation, likely due to limited diffusion of substrates and metabolites in the denser protein–polysaccharide matrix. Similar relationships were reported by Aljewicz et al. (2020) [19], who showed that oat β-glucan and curdlan, by modifying the microstructure of the gel, affected the transport of substrates and metabolites and the dynamics of lactose utilization. In turn, Brüls et al. (2024) [21] showed that different exopolysaccharides reorganize the casein network and modulate secondary sugar transformations by influencing gel mechanics and the susceptibility of the system to syneresis. Consistent with these findings, in the present study, higher doses of scleroglucan limited lactose metabolism, while the lowest level (0.25%) promoted more intensive hydrolysis and galactose accumulation.

### 2.4. Analysis of Volatile Compounds

The aroma of yogurt is the result of the microbial metabolism of lactose, proteins, and lipids by microorganisms, leading to the synthesis of compounds that give the product its characteristic sensory properties. Additional aroma notes may also be formed through interactions and transformations between volatile and non-volatile compounds [22]. The literature describes approximately 117 volatile compounds present in yogurt, including carbonyl compounds, acids, alcohols, esters, and other volatile metabolites [23]. The most important aroma-active compounds include acetaldehyde, diacetyl (2,3-butanedione), acetoin, 2,3-pentanedione, and acetic acid, which are the main components responsible for the sensory profile of yogurt [23].

**Figure 5:**
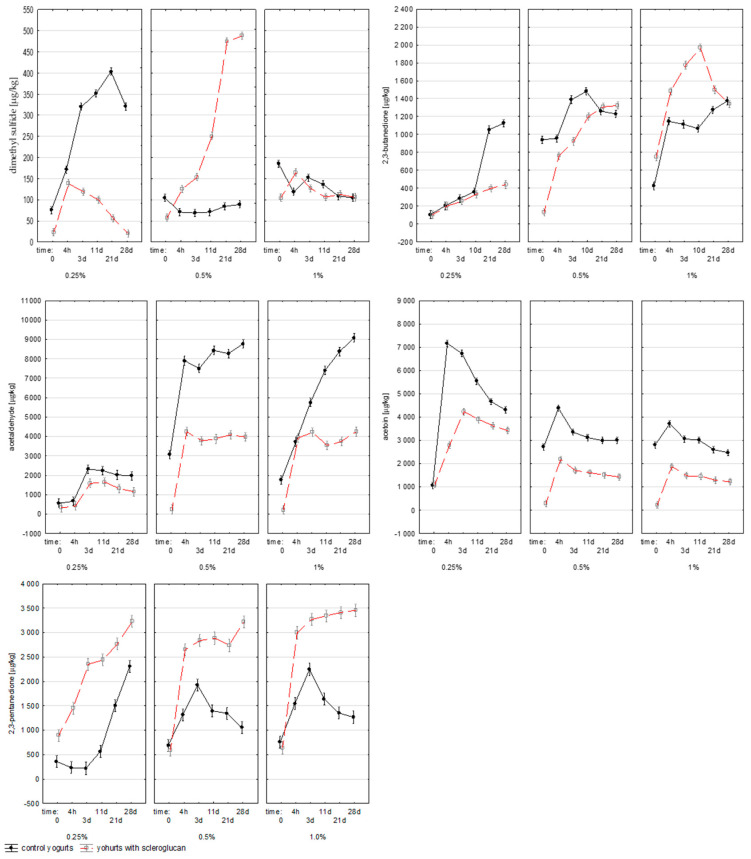
Average content of dimethyl sulfide, 2,3-butanodione, acetaldehyde, acetoin and 2,3 pentanedione (µg/1 kg) in milk (0), yogurt samples measured after fermentation (4 h) and after 3, 11, 21 and 28 days of refrigerated storage. Time 0 refers to raw milk before inoculation with starter cultures. The values 0.25%, 0.5%, and 1% indicate the concentration of scleroglucan in the yogurt samples. Data are presented as means (n = 3).

Dimethyl sulfide (DMS), derived from the degradation of sulfur-containing amino acids and lipid precursors, contributes a sweet, buttery aroma at low levels, but imparts undesirable sulfur notes at higher concentrations. Its synthesis by lactic acid bacteria is linked to the redox potential of the medium oxidative conditions favor DMS formation [24]. Due to its high specific surface area and greater susceptibility to oxidation, the addition of milk powder could generate additional sulfur precursors, increasing DMS accumulation [25]. Dimethyl sulfide (DMS) was strongly affected by both scleroglucan concentration (*p* < 0.001; η^2^p = 0.986) and fermentation time (*p* < 0.001; η^2^p = 0.969). As indicated in Figure 5, all samples exceeded the sensory threshold for DMS. The higher viscosity of scleroglucan-containing gels likely reduced oxygen diffusion, creating conditions favoring DMS accumulation.

Diacetyl (2,3-butanedione), a major carbonyl compound, plays a central role in shaping the buttery and creamy aroma of yogurt. Its accumulation was significantly influenced by milk powder content (*p* < 0.001; η^2^p = 0.985), fermentation time (*p* < 0.001; η^2^p = 0.980), and the interaction between factors (*p* < 0.001; η^2^p = 0.965). In the 0.25% C sample, the concentration exceeded 1.1 mg/kg after 28 d, while in yogurts with 0.25% S it was reduced to around 0.4 mg/kg, confirming the inhibitory effect of scleroglucan at low levels. Higher concentrations (0.5–1.0% S) resulted in a renewed increase, reaching up to 2.0 mg/kg. These patterns, as shown in Figure 5, indicated a concentration-dependent effect. All values were above the odor perception threshold (1.1 µg/kg), confirming the sensory relevance of diacetyl in all samples. In the literature, diacetyl values in yogurt range from 0.2–3.0 mg/kg, depending on the strains and fermentation conditions [26,27]. In conditions of limited carbon availability or in the presence of more slowly metabolized carbohydrates, lactic acid bacteria switch to mixed acid metabolism, producing a variety of products, including diacetyl and its derivatives [28]. Comparable relationships between fermentation conditions and diacetyl biosynthesis have been reported by Smid and Kleerebezem [29], and Irigoyen et al. [30]. The data presented show that scleroglucan had a significant effect on diacetyl biosynthesis, and the nature of this effect depended on the dose used.

Acetoin (3-hydroxy-2-butanone), formed by the reduction of diacetyl, provides mild, creamy notes that balance the acidic taste of yogurt. Its concentration depended significantly on milk powder level (*p* < 0.001; η^2^p = 0.974), fermentation time (*p* < 0.001; η^2^p = 0.977), and their interaction (*p* < 0.001; η^2^p = 0.971). In yogurts with scleroglucan, acetoin levels were generally lower, particularly during the early stages of fermentation. This may reflect a shift from reductive to oxidative metabolism associated with lower substrate mobility and oxygen transfer within a denser gel matrix. Similar metabolic competition between acetoin and diacetyl synthesis pathways has been described by Cheng et al. [28]. Reduced diffusion of substrates in a thicker gel matrix containing scleroglucan may have shifted the balance toward oxidative pathways, resulting in lower acetoin concentrations. Acetoin is responsible for the mild, creamy aftertaste that balances the pronounced sour notes of yogurt. Its lower levels in the scleroglucan samples may explain the milder sensory character of these products, which is confirmed by literature data indicating that other polysaccharides also limit the biosynthesis of short-chain carbonyl compounds (C_4_) [28].

Acetaldehyde is the main compound responsible for the fresh, characteristic aroma of yogurt, often described as “green apple” or “nutty.” It is produced by several metabolic pathways of *Lactobacillus* and *Streptococcus* species, including the conversion of lactose, valine, pyruvate, acetyl phosphate, and threonine [19,29]. In the control yogurts, the acetaldehyde concentration was strongly dependent on the milk powder level (*p* < 0.001; η^2^p = 0.989) and fermentation time (*p* < 0.001; η^2^p = 0.977). As can be seen in Figure 5, the 0.25% C variant reached about 2.3 mg/kg after fermentation, while in 0.5% C and 1.0% C, the concentrations were significantly higher, 8.8 and 9.1 mg/kg, respectively. Compared to the results of Aljewicz, et al. [19], where acetaldehyde levels were 5.1 mg/kg for skimmed yogurt and 0.63 mg/kg for full-fat yogurt, the values obtained in this study were markedly higher. These differences can be explained by the different fermentation conditions and increased oxygen diffusion resulting from higher milk powder content, which enhances precursor availability and redox activity. The reduced acidification rate observed in yogurts containing scleroglucan likely resulted from the diffusion-limiting properties of the polysaccharide matrix. The formation of a dense, triple-helical scleroglucan network restricted oxygen transfer and metabolite exchange, thereby altering the redox balance and pyruvate metabolism of lactic acid bacteria. This mechanism explains both the slower acidification and the lower acetaldehyde concentration observed in the experimental samples.

Figure 5 also illustrates that scleroglucan addition reduced acetaldehyde accumulation at 0.25% and 0.5% concentrations, confirming a similar inhibitory trend previously reported for oat β-glucan [19]. In yogurts with 1.0% scleroglucan, a rapid initial increase was observed, followed by a lower final concentration compared with the control. This behavior suggests that the polysaccharide may bind aldehyde precursors or modulate bacterial enzyme activity, thereby reducing the synthesis and accumulation of acetaldehyde during fermentation and storage.

2,3-Pentanedione, a diketone compound formed through the metabolism of branched-chain amino acids (valine and leucine) and pyruvate, is responsible for the buttery–creamy aroma characteristic of fermented milk products. In the control samples, the concentration initially decreased from 360.1 ± 8.3 µg/kg to 218.3 ± 8.0 µg/kg (3 d), followed by a marked increase to 1505.0 ± 42.6 µg/kg at 21 d and 2309.2 ± 138.5 µg/kg at 28 d. In yogurts with 0.25% scleroglucan, the content was higher throughout fermentation—from 893.8 ± 69.4 µg/kg initially to 2352.5 ± 139.6 µg/kg (3 d) and 3229.1 ± 129.4 µg/kg (28 d). Similar values were observed for 0.5% S and 1% S, reaching approximately 3000–3500 µg/kg. As evident in Figure 5, scleroglucan stimulated 2,3-pentanedione formation, most likely by enhancing oxidative carbon metabolism and altering carbon flow among pyruvate-derived pathways. The combined increase in 2,3-pentanedione and the selective decrease in diacetyl at low scleroglucan levels support this interpretation [29].

### 2.5. Microbiological Analysis—Viability of Starter Cultures

**Figure 6:**
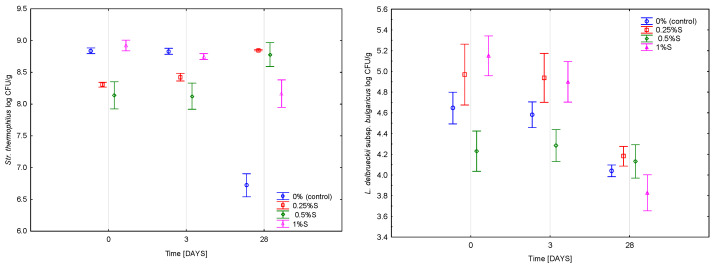
Average viability of starter cultures in yoghurt samples measured immediately after fermentation (0) and after 3^RD^ and 28^TH^ during 28 days of refrigerated storage. The values 0.25%, 0.5%, and 1% indicate the concentrations of scleroglucan in the yoghurt samples. Data are presented as mean values (n = 6), with error bars representing standard deviations.

The viability of *Streptococcus thermophilus* remained high throughout storage and showed similar levels across all samples up to day 21 (approx. 8.5 log CFU/g). As can be seen in Figure 6, a decline was observed in the control thereafter, reaching 5.85 log CFU/g by day 28, whereas the 1% S variant exhibited a smaller reduction of about 0.8 log unit. In contrast, the 0.5% S sample showed a slight increase (+0.65 log unit) between day 21 and day 28. Significant effects of scleroglucan concentration (*p* < 0.001; η^2^p = 0.351), storage time (*p* < 0.001; η^2^p = 0.217), and their interaction (*p* < 0.001; η^2^p = 0.941) were confirmed.

The viability of *Lactobacillus delbrueckii* subsp. *bulgaricus* was driven primarily by storage time (*p* < 0.001; η^2^p = 0.944), with a weaker effect of scleroglucan concentration (*p* < 0.001; η^2^p = 0.840). As illustrated in Figure 6, counts at day 28 reached approximately 4.3 log CFU/g in the control. A slight increase occurred in the 0.25% S variant (+0.1 log), whereas the 0.5% S sample showed a more pronounced decrease (0.67 log CFU/g). The 1% S variant remained comparable to the control.

These relationships are confirmed by the results of the experiments with pullulan [15] and previous studies concerning oat β-glucan and curdlan [19], which also demonstrated that polysaccharides can influence the survival dynamics of lactic acid bacteria by modifying the physical structure of the matrix and its diffusion properties. The reduction in *L. bulgaricus* counts was largely associated with the use of skimmed milk lacking the protective elements of the fat globule membrane, such as proteins and lipids. In this system, scleroglucan performed a sole stabilizing role, but its protective effect was limited compared to the natural components of the fat membrane [30]. The structural effects of scleroglucan such as increased viscosity, reduced serum mobility and a more compact matrix provided partial stabilization that was sufficient to maintain higher *S. thermophilus* counts but did not fully protect the more acid-sensitive *L. bulgaricus*. Overall, the results demonstrate that scleroglucan did not inhibit starter culture activity and, at higher concentrations, improved the late-stage stability of *S. thermophilus*, while exerting a more limited effect on *L. bulgaricus* due to the inherent constraints of a low-fat system.

### 2.6. Syneresis

In yogurts produced from skimmed milk, syneresis reached high levels. As shown in Table 2, after 3 days, whey separation amounted to 23.18% in the 0.25% C sample and 29.67% in the 1% C variant. After 28 days, syneresis decreased to approximately 20% in samples with higher milk powder content (0.5% C and 1% C), while no significant change was observed in the 0.25% C variant. As can be seen in Table 2, all scleroglucan-containing samples exhibited complete suppression of syneresis (0.00% at both 3 and 28 days).

These values in Table 2 indicate that the absence of syneresis in the scleroglucan variants directly reflects the microstructural changes observed in the SEM analysis. At 0.25–0.5%, the polysaccharide visibly smoothed and narrowed the pores within the protein network, while at 1.0% it produced a compact, continuous, nearly pore-free matrix. This structural reinforcement immobilized serum within the gel and prevented whey expulsion even during long-term storage.

In skimmed milk yogurts, the lack of fat globules weakens the structural integrity of the gel, making it highly susceptible to whey separation. Scleroglucan compensated for this deficit by integrating into the casein network, increasing its density and water-binding capacity, and reducing the size and connectivity of channels through which serum typically migrates. In control samples, the moderate reduction in syneresis by day 28 was associated with gradual restructuring of the protein matrix and improved hydration, particularly where higher levels of milk powder increased solids content. At lower solids, however, this effect was minimal due to insufficient network consolidation. The strong stabilising effect of scleroglucan corresponds with findings for other β-glucans and hydrocolloids, which enhance water retention through protein–polysaccharide interactions and the formation of denser gel structures [31,32,33,34]. Here, even the lowest concentration of scleroglucan (0.25%) eliminated whey separation, which is consistent with SEM observations and fully supported by the data in Table 2.

### 2.7. Rheology Analysis

The analysis of flow curves showed a good fit to the Herschel–Bulkley model (R^2^ = 0.897–0.998). As shown in Table 2, the coefficient of determination decreased systematically with increasing scleroglucan content and storage time, which can be attributed to increased cross-linking and the formation of stresses within the protein matrix. In the control samples, after mild mixing, the yield value (τ_0_) was close to zero, while the presence of scleroglucan caused a marked increase in this parameter: τ_0_ ≈ 16.04 Pa (0.25% S3), 25.24 Pa (0.5% S3), and 45.24 Pa (1% S3). As can be seen in Table 2, during storage, there was a slight decrease in the τ_0_ values (e.g., 0.25% S28, 0.5% S28, 1% S28), indicating a gradual reorganization of the protein–polysaccharide network while maintaining its stability. Scleroglucan in the triple-helical conformation increased the stiffness of the microstructure and limited the rate of its changes during storage. A similar effect was reported for other polysaccharides, where anionic gellan at a concentration of 0.02% caused an increase in τ_0_, improving yogurt texture [2,9].

The consistency coefficient (K) in control yogurts increased with the proportion of milk powder and storage time. As presented in Table 2, the response in scleroglucan variants was non-linear response: in the 0.25% S3 and 0.5% S3 samples, the K values were lower than in the controls (e.g., 0.25% C3: 115.51 Pa·s^n^ vs. 21.47 Pa·s^n^), while at 1% S addition, the index exceeded the control values (199.11 Pa·s^n^ vs. 156.60 Pa·s^n^). The increase in K over time was more pronounced in the 0.25% S and 0.5% S variants, indicating that the stabilization of the protein–polysaccharide network occurred gradually. At 1% scleroglucan, a high K value was observed after only 3 days, suggesting a faster attainment of microstructural equilibrium. The interactions responsible for these effects were non-covalent in nature (primarily hydrogen and hydrophobic interactions).

**Figure 7:**
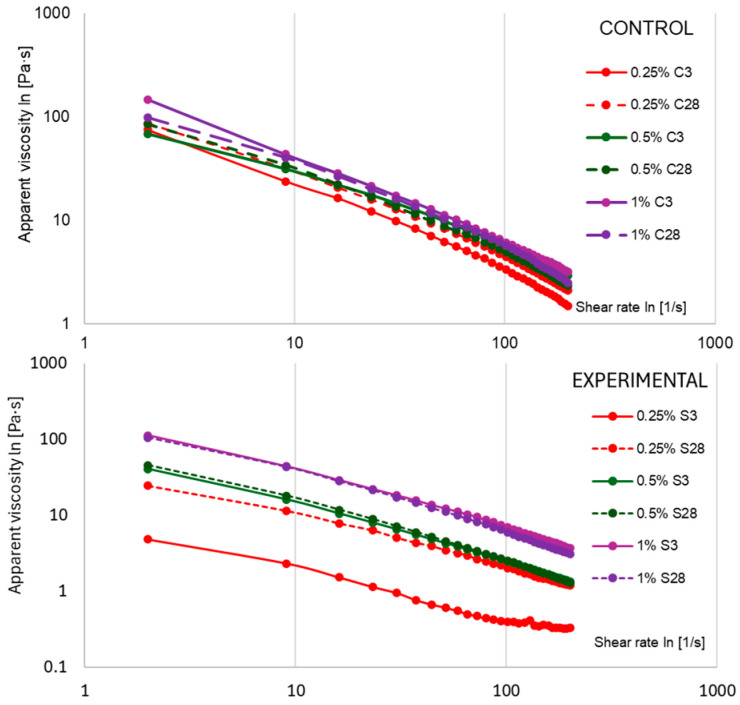
Average apparent viscosity of yogurt samples measured after 3 and 28 days of refrigerated storage. The values 0.25%, 0.5%, and 1% indicate the concentrations of scleroglucan in the experimental samples and skimmed milk powder in the control samples. Data are presented as means (n = 3).

The flow index (n) confirmed the increasing pseudoplasticity of yogurt samples during storage. As indicated by the values in Table 2, n remained low in the control samples and decreased further after 28 days (e.g., in 0.25% C from 0.101 to 0.064). Yogurts with scleroglucan exhibited higher n values after 3 days (in the 0.25% S and 0.5% S samples), reflecting a lower degree of pseudoplasticity compared to the controls. After 28 days, these values decreased (to 0.195 and 0.081, respectively), approaching the level of the control variants. These changes—an increase in the consistency coefficient (K) and yield point (τ_0_) with a simultaneous decrease in n values are characteristic of polysaccharide–stabilized systems [35]. As shown in Figure 7, the apparent viscosity (η) depended on both storage time and additive concentration. In variants with 0.25–0.5% S, η values at medium and high shear rates (50–100 s^−1^) were lower than in control samples, which facilitated flow in technological processes while maintaining stability at rest (τ_0_ > 0). In the 1% S samples, the η values exceeded the control values, resulting in a more pronounced sensation of density in the mouth and greater flow resistance, requiring adjustment of process parameters on an industrial scale (e.g., pump capacity).

### 2.8. Texture Analysis

As shown in Table 2, after 3 days of storage, the hardness of the control yogurts ranged from 1.58 N (0.25% C) to 2.09 N (1% C), while the samples containing scleroglucan showed higher values: 1.75 N (0.25% S), 2.25 N (0.5% S), and 5.34 N at 1% S. After 28 days, hardness increased slightly in all samples (C28: 2.33 N; S28: 3.43 N). The additive level remained the dominant factor determining texture (*p* < 0.001; η^2^p = 0.979).

The progressive increase in hardness with rising scleroglucan concentration corresponds with SEM observations, which showed that at 0.25–0.5% the polysaccharide partially filled the pores of the casein matrix, while at 1% it produced a compact, almost pore-free network. This structural reinforcement directly increased resistance to deformation during texture testing. This behavior reflects mechanisms reported for other hydrocolloids. Ge et al. (2022) demonstrated that even a very low concentration of gellan gum (0.02%) significantly increased yogurt hardness by strengthening the interactions within the protein matrix [2]. Similarly, Aljewicz et al. (2021) showed that β-glucan reorganized the casein network, increased gel density, and enhanced structural cohesion during acidification [9], which parallels the effects observed in the present study for scleroglucan. Zhao et al. (2020) reported that curdlan at concentrations of 0.10–0.50% formed its own gel network independent of casein, increased water-holding capacity and shear resistance, and markedly elevated hardness [36]. Scleroglucan exhibits a similar strengthening action, but achieves it at lower concentrations due to the formation of a hydrated, triple-helical network that integrates efficiently with casein micelles.

The values presented in Table 2 also show that adhesiveness decreased across all samples as storage progressed, ranging from −14.13 N·s (0.25% C3) to −3.64 N·s (1% C3), and from −23.67 N·s (0.25% S3) to −6.92 N·s (1% S3). This reduction resulted from the formation of a tighter, more cohesive protein–polysaccharide network that limited free surface moisture and reduced stickiness. The texture behavior aligns strongly with rheological properties: scleroglucan increased the yield stress and consistency coefficient while decreasing the flow index. The concurrent rise in τ_0_ and K values, combined with SEM confirmed network compaction, explains the observed increase in hardness and the reduction in adhesiveness.

### 2.9. Microstructure Analysis

**Figure 8:**
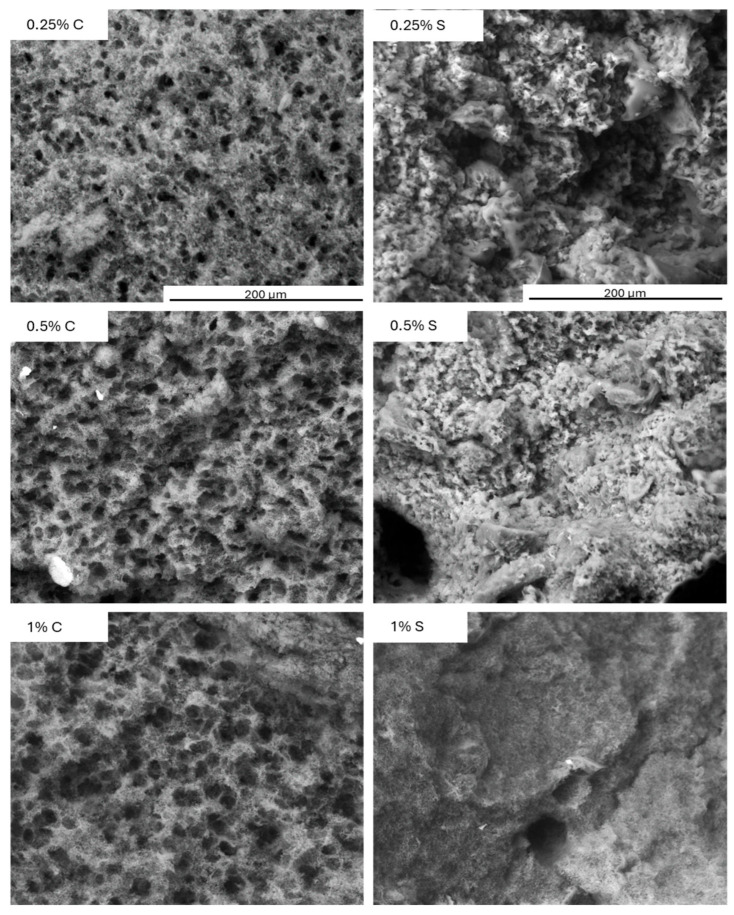
Microstructure analysis of control (C) and experimental (S) yogurt samples containing scleroglucan after 3 days of refrigerated storage. Magnification: 400×. Color analysis and visual properties.

The microstructure of the protein network in the control yogurt samples was compact yet porous, which was due to the absence of a natural filler, namely fat. As can be seen in Figure 8, the addition of scleroglucan led to distinct microstructural changes: at lower concentrations (0.25–0.5%), partial smoothing of the network was observed, while at 1.0%, the structure became compact and virtually free of pores.

The presence of the polysaccharide was visible throughout the protein network, consistent with the previously obtained textural and rheological results. As the concentration of scleroglucan increased, there was an increase in hardness, yield point, and consistency coefficient, and a decrease in the flow index. These changes indicate that the polysaccharide reinforced and compacted the protein network. Similar relationships have been demonstrated in previous studies [37], where oat β-glucan increased water-holding capacity and improved sensory properties by modifying the microstructure. Zhao et al. (2020) [36] noted that curdlan, thanks to its ability to form a gel, produced a denser and more resistant protein network. In the present study, the reduction of syneresis and improved color uniformity were a direct consequence of the microstructural sealing effect visible in Figure 8.

This phenomenon is also confirmed in studies on processed cheeses, where the presence of this polysaccharide led to the formation of a smooth and homogeneous surface. The mechanism has been attributed to the incorporation of scleroglucan into the protein matrix [38].

### 2.10. Color Analysis and Visual Properties

As shown in Table 2, the CIELAB color parameters demonstrated that the effect of scleroglucan on yogurt color was indirect and largely associated with microstructural differences within the gel. Yogurts containing the polysaccharide were darker and less yellow than the corresponding control samples. At 0.25% S, the total color difference reached ΔE = 6.81, while at 0.5% S and 1% S the values were ΔE = 12.03 and ΔE = 4.21, respectively. In the control samples, ΔE decreased with increasing milk powder content (0.25% C: 4.84; 0.5% C: 1.23; 1% C: 0.27). After 28 days, the greatest deviation was observed for the 0.25% S variant (ΔE = 18.98; ΔL = −18.83; Δa = +0.05; Δb = −2.41), whereas the 0.5% S and 1% S samples showed only minimal changes (ΔE = 1.31 and 0.51).

These values in Table 2 indicate that the optical properties correspond closely with the SEM findings. At low concentrations, scleroglucan produced an uneven, partially compacted network that scattered light irregularly, resulting in lower L values and greater ΔE. At higher levels, the matrix became more uniform and densely structured, limiting internal scattering and stabilizing color during storage. These relationships confirm that the visual appearance of yogurt was governed primarily by gel homogeneity and water distribution rather than direct pigment-related effects.

These findings are consistent with previous observations for other stabilizers used in fermented dairy systems. Both xanthan and guar gum have been shown to improve optical homogeneity by reducing syneresis and reinforcing the protein network [39]. Inulin increases uniformity at sufficient concentrations by thickening the aqueous phase and filling the pores in the casein matrix, despite not altering L, a or b* values directly [18]. Pullulan enhances visual smoothness through improved gel stability, although it does not significantly influence color parameters [15]. In the present study, the visual stability of the 0.5% S and 1% S samples reflects the formation of a coherent and well-integrated protein–polysaccharide matrix, consistent with textural and rheological results. Only the 0.25% S variant showed clear deviations, attributable to incomplete microstructural cross-linking and heterogeneous light scattering. From a consumer perspective, the perceptibility thresholds indicate that ΔE values below 2.0 remain barely noticeable, which was achieved only in the samples containing 0.5% and 1% scleroglucan.

### 2.11. Sensory Evaluation and Overall Acceptability

Table 3 shows the average values of sensory attributes of control yogurts and yogurts enriched with scleroglucan. The attributes were classified according to appearance, aroma, consistency, mouthfeel, and taste, as well as overall acceptability. The evaluation was carried out on the tenth day of storage, i.e., at a different point in time than the instrumental measurements taken after 3 and 28 days and the analyses of volatile compound profiles conducted throughout the storage period. The sensory evaluation results were largely consistent with the instrumental analyses, with a few differences observed, which were due to the differences in the methods used.

As shown in Table 3, all yogurts were characterized by very high color uniformity (*p* > 0.05). The cream color was higher in the control yogurts and lower in the variants with scleroglucan (*p* < 0.05). As can be seen in Table 3, this trend was confirmed by the CIELAB color parameters which showed lower L* and b* values in the scleroglucan-containing samples and higher ΔE values, particularly at 0.25% S. Significant whey leakage occurred in the control yogurts, while this phenomenon did not occur in the experimental samples, according to the syneresis measurements (0% S at days 3 and 28).

Aroma. The values in Table 3 indicate that the yogurt aroma was particularly intense in the control samples and decreased with the addition of scleroglucan, as did the sour aroma (*p* < 0.05). The lowest values for both attributes were recorded at 1% S, which reflected lower acidity (°SH values), slower acidification, and less accumulation of acetaldehyde in the polysaccharide variants. No sweet or foreign odors were detected. The increase in diketones (2,3-pentanedione, diacetyl in certain variants) in the scleroglucan samples may have enhanced the creamy–buttery notes, which were not identified as a separate attribute.

Consistency. The patterns in Table 3 demonstrate that uniformity in control yogurts decreased with the addition of SMP, while in experimental variants it increased with polysaccharide concentration. Lumpiness was highest at 0.25% S, and the highest “thickness” scores were obtained in 1% S yogurts (*p* < 0.05). The sensory evaluation of thickness and uniformity was consistent with the textural and rheological parameters, as the highest density values in the 1% S variant corresponded to simultaneously high values of hardness, yield point (τ_0_), and consistency coefficient (K). However, the sensory panel indicated high lumpiness at 0.25% S, which was consistent with microstructure analyses showing incomplete gel cross-linking and greater heterogeneity.

Mouthfeel. Adhesiveness, understood as the tendency of the yogurt to adhere to the palate, was scored as moderate in all variants, with the highest values found at 1% S. As indicated in Table 3, the difference between the adhesiveness values measured by the texture profile analysis and the panelists’ perception was due to the different nature of both methods. The instrumental measurements reflected the force required to detach the probe, while the sensory evaluations were more related to the high yield point (τ_0_) and viscosity at low shear rates (η = 10 s^−1^), which were elevated in the variants with 1% S. For this reason, these yogurts were perceived as more adhesive, despite lower adhesiveness values obtained in the instrumental tests.

Taste. The intensity of the typical yogurt taste and sour taste was the lowest for the 1% S samples. This phenomenon reflected lower acidity values and reduced acetaldehyde production. Sweetness reached higher values at 1% S (*p* = 0.001), which can be explained by a milder acid profile and increased thickness, without a significant increase in the concentration of simple sugars. Bitter and foreign tastes were not detected. The sensory panel scored the overall acceptability of all samples. According to Table 3, the lowest scores were given to yogurts with 0.25% S and 0.5% S, which were associated with a perceived mealy mouthfeel. The control yogurts were scored as average—they had the most typical yogurt taste but were less thick and had the highest whey leakage. The highest scores were given to yogurt with 1% S, which, according to the panelists, had the mildest aroma, was the thickest, and had a smooth consistency.

## 3. Materials and Methods

### 3.1. Materials

Low-fat cow’s milk (0.5% fat) was obtained from a local dairy plant (Olsztyn, Poland). A commercial yogurt starter culture (YC-180, Chr. Hansen, Denmark) containing *Streptococcus thermophilus* and *Lactobacillus delbrueckii* subsp. *bulgaricus* was used for fermentation. Experimental yogurts were produced with the addition of highly purified β-glucan (scleroglucan, 90%; Mw = 0.97 × 10^6^ g/mol) isolated from *Sclerotium rolfsii* (Cargill, Krefeld, Germany). Skimmed milk powder (SMP) (Mlekpol, Mrągowo, Poland) was applied in the standardization process. All chemicals and reagents were of analytical grade and were obtained from Sigma-Aldrich (St. Louis, MO, USA).

### 3.2. Experiment Design

Two sets of data were collected. The first pertained to the yogurt production process. Time 0 h corresponds to the moment before the starter culture was added to the milk. The 4 h point corresponds to the end of fermentation and the achievement of a pH of 4.6. The second set of data covered the storage period of the finished yogurt. In this case, the value 4 h marked the beginning of storage. Samples were stored at 4 ± 1 °C throughout the storage period. The results were limited to the third day (Day 3), which corresponds to the stabilization of the structure, and to Day 28, which is the end of the shelf life. The sensory evaluation is an exception. It was performed on Day 21 of storage, which corresponds to the average time the yogurt spends on the store shelf.

### 3.3. Yogurt Production

Yogurts were produced under laboratory conditions following the Polish patent PL235801. The milk was heated to 45 °C, and the required amount of scleroglucan (0.25–1.0% *w*/*w*) or SMP (control) was added. The mixture was stirred at 45 °C for 30 min to ensure complete hydration of dry ingredients. The milk was then pasteurized at 90 °C for 10 min and cooled to 43 °C before inoculation. The starter culture (0.02 g/100 g) was added, and the samples were poured into 100 mL sterile containers, covered, and incubated at 43 °C until pH 4.6 (approximately 4 h). After fermentation, yogurts were cooled to 4 °C and stored for subsequent analyses. Packaging: 100 mL cups (Ø57 × H76 mm). Separate cups were used for each analysis. To ensure comparability between samples, the milk base was standardized with respect to total solids. The amount of skim milk powder added to the control variants corresponded to the dry-matter contribution of scleroglucan in the experimental samples. This approach ensured that all yogurts were produced from a uniform raw material and ensured that differences observed between variants reflected the effect of scleroglucan rather than variation in overall solids content.

### 3.4. Microbiological Analysis

Enumeration of *Streptococcus thermophilus* was performed on M17 agar (Merck, Darmstadt, Germany), with plates incubated aerobically at 45 °C for 48 h. Viable counts of *Lactobacillus delbrueckii* subsp. *bulgaricus* were determined on Rogosa agar (Merck), incubated anaerobically at 37 °C for 72 h using the AnaeroGen system (Oxoid, UK).

### 3.5. The Acidification Activity

Acidification activity was monitored with a multi-channel pH/pC/mV multiplexer (Cerko, Gdynia, Poland) equipped with ERH-13-6 electrodes (Hydromet, Gliwice, Poland). The initial pH of the milk was recorded at 43 °C, and the fermentation endpoint was set at pH 4.6. Acidification curves (pH vs. time) were recorded every 1 min, and the time required to reach pH 4.6 was defined as the acidification time (t_4.6_). The titratable acidity (°SH) was determined by titrating 10 g of the sample with 0.1 mol/L NaOH using phenolphthalein as an indicator. Results were expressed as Soxhlet–Henkel degrees (°SH).

### 3.6. Isolation of Volatiles

Volatile compounds were analyzed according to the method of Aljewicz et al. (2020) [19]. For each analysis, 14 g of the sample was placed in 20 mL vials and spiked with isotopically labeled internal standards to a final concentration of 300 ppb. Volatiles were extracted from the headspace by solid-phase microextraction (SPME) using a CAR/PDMS fiber (Supelco, Bellefonte, PA, USA) at 50 °C for 5 min, followed by 45 min fiber exposure and desorption at 260 °C. Analyses were performed using a GC × GC-ToF-MS system (Pegasus IV, LECO Corporation, St. Joseph, MI, USA) equipped with an SPB-5 primary column and a Supelcowax 10 secondary column, operating with a helium flow of 0.8 mL/s. Compounds were identified by comparison with the NIST mass spectral library and by retention indices calculated relative to a C7–C20 n-alkane series. Quantification was based on analyte-to-standard peak area ratios using ChromaTOF software. For selected compounds, a stable isotope dilution assay (SIDA) was applied, with quantitation ions and isotopically labeled standards summarized in Table 4. Labeled internal standards: D_6_-acetone, D_8_-toluene, D_4_-acetaldehyde, D_4_-2,3-butanedione, and D_6_-acetoin were used in the GC–MS analyses. Results were expressed as µg/kg of yogurt.

### 3.7. Lactic Acid and Citrate Analysis

Lactic and citric acids were quantified by HPLC following a modified method of Aljewicz et al. (2020) [19]. Samples (1 mL) were diluted in 0.05 M H_2_SO_4_, vortexed, incubated at 40 °C, centrifuged, and filtered (0.45 µm RC, Sigma-Aldrich). Aliquots (20 µL) were analyzed in an Agilent 1260 HPLC system with a DAD detector at 215 nm. Separation was performed on a Supelcosil LC-610H cation-exchange column (300 × 7.8 mm) with H^+^ guard at 60 °C, using 0.05 M H_2_SO_4_ as the mobile phase (0.5 mL/min). Lactic and citric acids were identified against authentic standards and quantified by external calibration (R^2^ > 0.99). Note: In milk, citric acid is predominantly present as citrate anions. Results were expressed as g/kg of yogurt.

### 3.8. Carbohydrate Analysis

Carbohydrates were analyzed according to Aljewicz et al. (2020) [19], with minor modifications. Samples (1 mL) were diluted, vortexed, incubated at 40 °C, centrifuged, and filtered (0.45 µm RC, Sigma-Aldrich). Aliquots (20 µL) were injected into an Agilent 1260 HPLC system (Agilent Technologies, Santa Clara, CA, USA) with ELSD detection (50 °C, 3.5 bar). Separation was achieved on a Supelcosil LC-610H cation-exchange column (300 × 7.8 mm) with an H^+^ guard cartridge at 30 °C, using Milli-Q^®^ (Merck, Darmstadt, Germany)water as the mobile phase (1 mL/min). Carbohydrates were identified by retention times of authentic standards and quantified by external calibration (R^2^ > 0.99).

### 3.9. Microstructure Analysis

The microstructure analysis was performed according to Aljewicz et al. (2024) [40] using SEM (Quanta 200, FEI Company, Hillsboro, OR, USA). Samples were fixed in 2.5% glutaraldehyde, dehydrated in graded ethanol (30–100%), and dried at the critical point. Specimens were sputter-coated with gold and observed at 400× magnification, with a 30 kV accelerating voltage under high vacuum.

### 3.10. Rheological Properties

Rheological characteristics of the produced yogurts were determined using a Rheolab QC rotational rheometer (Anton Paar, Graz, Austria) equipped with a coaxial cylinder system (CC39-SN46605, CC27/QC-LTD). Apparent viscosity and shear stress were measured across a shear rate range of 1–100 s^−1^ at 10 °C, with three replicates per product after gentle mixing, using the RHEOPLUS/32 V3.40 software (Rheolab QC manual). Results were expressed as flow curves (shear stress vs. shear rate). To describe the rheological behavior, the experimental data were fitted to the Herschel–Bulkley model, which showed the highest coefficient of determination (R^2^). This model is commonly applied to yogurts and other gel-like dairy systems because of their yield stress behavior.

The model is expressed as:

(1) $$\tau =\tau_{0}+Ƙ\cdot {\dot{\gamma }}^{n}$$

where: τ—shear stress (Pa), $\tau_{0}$—yield stress (Pa), representing the minimum stress required to initiate flow, Ƙ—consistency coefficient/index (Pa∙sn), describing the viscosity of the fluid, $\dot{\gamma }$—shear rate (s^−1^), n—flow behavior index, indicating whether the fluid is shear-thinning (n < 1), Newtonian (n = 1), or shear-thickening (n > 1).

Apparent viscosity values were compared at selected shear rates: 10 s^−1^, corresponding to conditions similar to mastication forces during food consumption; 50 s^−1^, often associated with oral processing and swallowing; and 100 s^−1^, reflecting flow behavior in industrial processing and pumping systems.

### 3.11. Texture Analysis

The texture analysis was performed according to Aljewicz et al. (2024) [40]. Texture was assessed using a TA-XT2i analyzer (Stable Micro Systems, UK) with a 10 mm stainless-steel cylinder probe (P/10). Samples were stored at 4 °C for 24 h and equilibrated for 30 min at 20–22 °C prior to testing. The probe penetrated samples at 1 mm/s to a depth of 30 mm, and force was recorded with Exponent v6.2. Firmness and adhesiveness were determined (n = 6 per sample).

### 3.12. Syneresis of Yogurt

Syneresis was determined by the centrifugation method. Yogurt samples (21 g) were placed in 50 mL centrifuge tubes and centrifuged for 15 min at 10 °C and 3000× *g* using a Heraeus Megafuge 16R (Thermo Scientific, Osterode am Harz, Germany) with a TX-400 rotor (Rmax 168 mm). The separated whey was weighed, and the results were expressed as a percentage of expelled serum (% syneresis).

### 3.13. Sensory Analysis

Control yogurts with added skimmed milk powder (SMP) and experimental yogurts with added scleroglucan were subjected to sensory evaluation using the profiling method according to PN-EN ISO 13299:2016-05E [41]. The study was conducted by an eight-member sensory panel trained in the evaluation of dairy products, consisting of experienced academic staff in the sensory analysis laboratory. The panelists had appropriate sensory sensitivity in accordance with PN-EN ISO 8586:2012 [42]. Based on definition sheets, the intensity of 18 sensory attributes and the overall acceptability of the yogurts were evaluated. A five-point descriptive scale was used, where 1 indicated the absence of a given attribute and 5 indicated very strong intensity. Samples were coded and evaluated on the tenth day after production. According to Polish legislation, approval from a bioethics committee is not required for sensory evaluation studies involving food products.

### 3.14. Color Measurement

Color parameters (L* (lightness), a* (red–green coordinate), and b* (yellow–blue coordinate)) were determined using a Konica Minolta CM-5 colorimeter (Tokyo, Japan) calibrated with a white standard plate. The total color difference (ΔE*) between samples and the control was calculated as:

(2) $$∆E*=\sqrt{{\left(∆L*\right)}^{2}+{\left(∆a*\right)}^{2}+{\left(∆b*\right)}^{2}}$$

Color differences were classified as: ΔE* < 1.0—imperceptible; 1.0 ≤ ΔE* ≤ 2.0—slight; 2.0 ≤ ΔE* ≤ 3.0—noticeable; ΔE* > 3.0—clearly perceptible.

### 3.15. Statistical Analysis

Data were analyzed using Statistica 13.3 (TIBCO Software Inc., Palo Alto, CA, USA). A two-way ANOVA (factors: scleroglucan concentration and storage time) was applied, followed by Tukey’s post hoc test (*p* < 0.05). Effect sizes were reported as partial eta squared (η^2^p. When assumptions of normality or homogeneity of variance were violated, the Kruskal–Wallis test with ε^2^ effect size was used. Data from the sensory evaluation of yogurts were analyzed using one-way ANOVA and Fisher’s NIR post hoc test. Results are expressed as mean ± standard deviation (SD). The experiment was conducted twice (two independent production series considered as biological replicates). Each analysis within a series was performed in triplicate (technical replicates), yielding a total of n = 6 measurements per treatment. This design ensured reproducibility and provided adequate statistical power for subsequent analyses.

## 4. Conclusions

Scleroglucan significantly influenced the properties of yogurt made from skimmed milk, modifying both fermentation kinetics and the structural characteristics of the final product. Its addition slowed acidification, improved gel stability, and completely eliminated syneresis, while increasing hardness and viscosity, particularly at higher concentrations. Microstructural analysis confirmed enhanced protein network organization and water retention, resulting in greater homogeneity and improved sensory quality. The modification of volatile compound profiles—characterized by reduced acetaldehyde and elevated diketone levels—contributed to a milder and more balanced flavor.

Overall, scleroglucan at 1.0% effectively stabilized low-fat yogurt by improving viscosity, texture, and microbial viability without impairing fermentation. Its neutral charge and triple-helical conformation provide a distinct structural advantage over other β-glucans, making it a promising clean-label stabilizer for industrial applications in low-fat fermented dairy products.

## Figures and Tables

**Table 1:** Chemical composition of yogurt samples containing 0.25%, 0.5%, and 1% additives. Control samples were prepared with skimmed milk powder (SMP), while experimental samples contained scleroglucan at corresponding concentrations. Data are presented as means ± standard deviation (SD; n = 3).

		0.25%	0.5%	1%
CONTROL	DRY MATTER [%]	12.63 ^c^ ± 0.02	13.50 ^a^ ± 0.02	13.01 ^b^ ± 0.02
	FAT [%]	0.06 ^a^ ± 0.00	0.06 ^a^ ± 0.00	0.05 ^a^ ± 0.00
	PROTEIN [%]	4.53 ^a^ ± 0.01	3.98 ^b^ ± 0.01	4.51 ^a^ ± 0.00
EXPERIMENTAL	DRY MATTER [%]	12.89 ^c^ ± 0.03	13.82 ^b^ ± 0.02	14.17 ^a^ ± 0.06
	FAT [%]	0.05 ^b^ ± 0.00	0.06 ^a^ ± 0.00	0.07 ^a^ ± 0.00
	PROTEIN [%]	4.47 ^b^ ± 0.01	3.97 ^c^ ± 0.02	4.50 ^a^ ± 0.00

a–c—mean values in rows marked with different letters differ significantly at *p* ≤ 0.05.

**Table 2:** Functional properties of control (C) and experimental (S) yogurt samples measured after 3 and 28 days of refrigerated storage. S denotes yogurts containing scleroglucan. Rheological properties: HB—Herschel–Bulkley model; yield stress (τ_0_), apparent viscosity (η) at shear rates of 10, 50, and 100 s^−1^; flow behavior index (n); and consistency coefficient (K). Results are expressed as means (n = 3). For syneresis, texture, and color parameters, data are presented as means ± standard deviation (SD; n = 3).

	0.25%				0.5%				1%			
	C3	C28	S3	S28	C3	C28	S3	S28	C3	C28	S3	S28
Rheology properties												
R^2^ HB	0.995	0.988	0.979	0.989	0.995	0.994	0.998	0.996	0.935	0.939	0.945	0.897
HB tau0	1.75 × 10^−11^	1.40 × 10^−9^	1.60 × 10^1^	1.51 × 10^1^	1.89 × 10^−13^	5.14 × 10^−14^	2.52 × 10^1^	2.43 × 10^1^	2.20 × 10^−11^	2.31 × 10^−14^	4.52 × 10^1^	4.43 × 10^1^
HB k	115.515	122.039	21.466	42.463	134.956	170.247	28.490	87.387	156.600	176.413	199.108	211.745
HB n	0.101	0.064	0.874	0.195	0.159	0.073	0.224	0.081	0.145	0.054	0.124	0.077
η at 10 [Pa·s]	23.800	31.500	2.340	11.500	31.300	34.300	16.200	18.200	43.300	40.300	44.400	43.700
η at 50 [Pa·s]	6.240	8.320	0.612	3.460	9.830	8.740	4.300	4.600	11.200	10.300	12.400	11.200
η at 100 [Pa·s]	3.330	4.470	0.395	2.040	5.720	4.900	2.500	2.550	6.110	5.700	7.060	6.050
Syneresis [%]												
Syneresis [%]	23.18 ± 1.69	25.23 ± 2.33	0.00 ± 0.00	0.00 ± 0.00	22.19 ± 0.75	25.80 ± 0.94	0.00 ± 0.00	0.00 ± 0.00	29.67 ^a^ ± 1.15	20.03 ^b^ ± 0.69	0.00 ± 0.00	0.00 ± 0.00
Texture properties												
Hardness [N]	1.58 ^b^ ± 0.10	2.05 ^a^ ± 0.10	1.75 ^b^ ± 0.26	2.58 ^a^ ± 0.19	1.85 ^b^ ± 0.08	2.45 ^a^ ± 0.07	2.25 ^b^ ± 0.11	2.64 ^a^ ± 0.15	2.09 ^b^ ± 0.08	2.48 ^a^ ± 0.08	5.34 ^b^ ± 0.75	5.06 ^a^ ± 0.03
Adhesiveness [NxSEC]	−14.13 ^b^ ± 2.35	−9.34 ^a^ ± 0.98	−23.67 ± 9.78	−13.50 ± 1.06	−6.31 ± 1.26	−4.46 ± 1.03	−13.38 ± 5.13	−23.25 ± 7.72	−3.64 ± 1.27	−3.31 ± 1.63	−6.92 ^a^ ± 1.87	−8.99 ^b^ ± 0.75
Colour in CIELAB												
L	78.50 ^b^ ± 0.18	83.23 ^a^ ± 0.02	72.23 ^a^ ± 0.16	53.41 ^b^ ± 0.24	83.86 ^a^ ± 0.02	82.63 ^b^ ± 0.05	71.97 ± 0.09	70.71 ± 0.20	85.12 ± 0.27	84.98 ± 0.18	80.92 ± 0.24	80.53 ± 0.11
a	−2.30 ^a^ ± 0.05	−2.75 ^b^ ± 0.01	−3.59 ± 0.04	−3.54 ± 0.04	−2.70 ± 0.03	−2.77 ± 0.03	−2.82 ^a^ ± 0.03	−3.16 ^b^ ± 0.01	−2.58 ^a^ ± 0.07	−2.73 ^b^ ± 0.03	−2.61 ^a^ ± 0.02	−2.76 ^b^ ± 0.02
b	6.39 ^b^ ± 0.05	7.31 ^a^ ± 0.02	4.04 ^a^ ± 0.23	1.64 ^b^ ± 0.12	6.93 ± 0.01	6.84 ± 0.08	5.10 ± 0.04	5.23 ± 0.04	7.23 ± 0.10	7.05 ± 0.19	6.93 ± 0.25	7.24 ± 0.09

a–b—mean values in rows marked with different letters differ significantly at *p* ≤ 0.05; No index means that the results do not differ significantly.

**Table 3:** Average values of sensory attributes of control (C) and experimental (S) yogurt samples containing scleroglucan, evaluated after 10 days of refrigerated storage. Sensory attributes were classified into appearance, aroma, consistency, mouthfeel, taste, and overall acceptability.

Sensory Attibutes	0.25% C	0.5% C	1% C	0.25% S	0.5% S	1% S	*p*-Value
APPEARANCE							
uniform	4.5	4.6	4.6	4.7	4.7	4.7	>0.05
creamy colour	3.1 ^a^	3.1 ^a^	3.0 ^a^	2.5 ^b^	2.5 ^b^	2.4 ^b^	0.001
whey separation	3.7 ^a^	3.5 ^a^	3.5 ^a^	1.1 ^b^	1.0 ^b^	1.0 ^b^	0.000
AROMA							
typical for yoghurt	4.6 ^a^	3.9 ^b^	3.9 ^b^	3.2 ^c^	2.8 ^d^	2.5 ^d^	0.000
sour	3.6 ^a^	3.6 ^a^	3.4 ^a^	2.7 ^b^	2.5 ^b^	2.1 ^c^	0.000
sweet	1.0	1.0	1.0	1.0	1.0	1.0	>0.05
atypical	1.0	1.0	1.0	1.0	1.0	1.0	>0.05
CONSISTENCY							
uniform	3.9 ^a^	3.6 ^b^	3.5 ^b^	2.4 ^c^	3.5 ^b^	3.9 ^a^	0.000
lumpy	2.2 ^b^	3.4 ^a^	3.4 ^a^	3.7 ^a^	1.8 ^c^	1.5 ^c^	0.000
thick	1.5 ^d^	1.8 ^d^	2.5 ^c^	3.1 ^b^	3.2 ^b^	4.4 ^a^	0.000
MOUTH FEEL							
adhesive	2.3 ^b^	2.4 ^b^	2.5 ^b^	2.3 ^b^	2.6 ^b^	3.0 ^a^	0.000
smooth	3.1 ^b^	2.7 ^c^	2.4 ^c^	1.4 ^d^	1.6 ^d^	4.3 ^a^	0.004
mealy	1.5 ^c^	1.6 ^bc^	1.9 ^b^	3.9 ^a^	3.7 ^a^	1.3 ^c^	0.000
TASTE							
typical for yoghurt	4.5 ^a^	4.4 ^a^	4.2 ^a^	2.6 ^b^	2.5 ^b^	2.0 ^c^	0.000
sour	3.9 ^a^	3.8 ^a^	3.6 ^a^	2.8 ^b^	2.7 ^b^	2.1 ^c^	0.000
sweet	1.1 ^b^	1.1 ^b^	1.3 ^b^	1.4 ^b^	1.4 ^b^	1.8 ^a^	0.001
bitter	1.0	1.0	1.0	1.0	1.0	1.0	>0.05
atypical	1.0	1.0	1.0	1.0	1.0	1.0	>0.05
OVERALL ACCEPTABILITY	3.7 ^b^	3.5 ^b^	3.6 ^b^	2.1 ^c^	2.3 ^c^	4.5 ^a^	0.003

a–d—mean values in rows marked with different letters differ significantly at *p* ≤ 0.05.

**Table 4:** Labeled standards and quantitation ions used for SIDA (stable isotope dilution assay).

Compound	Quant. Ions (m/z) ^a^	Labeled Standards	Ion IS (m/z) ^b^
Dimethyl sulfide	62	^2^H_6_ Dimethyl sulfide	68
2,3-Butanedione	86	^13^C_4_ 2,3-Butanedione	90
Acetaldehyde	44	^2^H_4_ Acetaldehyde	48
2,3-Pentanedione	100	^13^C_4_ 2,3-Butanedione	90
Acetoin	88	^13^C_4_ 2,3-Butanedione	90

^a^—Ions of analytes used for quantitation, ^b^—ions of internal standards (labeled isotopes) used for quantitation.

## Data Availability

The original contributions presented in the study are included in the article, further inquiries can be directed to the corresponding author.

## References

[1] Jaman S., Islam M.Z., Sojib M.S.I., Hasan M.S., Khandakar M.M.H., Bari M.S., Sarker M.A.H., Habib R., Siddiki M.S.R., Islam M.A. (2022). Physicochemical Characteristics, Sensory Profile, Probiotic, and Starter Culture Viability of Synbiotic Yogurt. J. Adv. Vet. Anim. Res..

[2] Ge Z., Yin D., Li Z., Chen X., Dong M. (2022). Effects of Commercial Polysaccharides Stabilizers with Different Charges on Textural, Rheological, and Microstructural Characteristics of Set Yoghurts. Foods.

[3] Rafiq L., Zahoor T., Sagheer A., Khalid N., Rahman U.U., Liaqat A. (2020). Augmenting Yogurt Quality Attributes through Hydrocolloidal Gums. Asian-Australas. J. Anim. Sci..

[4] Kalab M., Emmons D.B., Sargant A.G. (1975). Milk-Gel Structure. IV. Microstructure of Yoghurts in Relation to the Presence of Thickening Agents. J. Dairy Res..

[5] Sinangil Z., Taştan Ö., Baysal T. (2022). Beta-Glucan as a Novel Functional Fiber: Functional Properties, Health Benefits and Food Applications. Turk. J. Agric.-Food Sci. Technol..

[6] Mejri W., Bornaz S., Sahli A. (2014). Formulation of Non-Fat Yogurt with β-Glucan from Spent Brewer’s Yeast. J. Hyg. Eng. Des..

[7] Antontceva E., Belyakova T., Zabodalova L., Shamtsyan M. (2019). Fortification of Yogurt with β-Glucans from Oyster Mushroom. Food Nutr. Well-Being.

[8] Kaur R., Riar C.S. (2020). Sensory, Rheological and Chemical Characteristics during Storage of Set Type Full Fat Yoghurt Fortified with Barley β-Glucan. J. Food Sci. Technol..

[9] Aljewicz M., Mulet-Cabero A.-I., Wilde P.J. (2021). A Comparative Study of the Influence of the Content and Source of β-Glucan on the Rheological, Microstructural Properties and Stability of Milk Gel during Acidification. Food Hydrocoll..

[10] Du B., Meenu M., Liu H., Xu B. (2019). A Concise Review on the Molecular Structure and Function Relationship of β-Glucan. Int. J. Mol. Sci..

[11] Brigand G. (1993). SCLEROGLUCAN. Industrial Gums.

[12] Sletmoen M., Stokke B.T., Geissler E. (2006). Small Angle X-Ray Scattering Study of Local Structure and Collapse Transition of (1,3)-β-D-Glucan-Chitosan Gels. J. Chem. Phys..

[13] Moresi M., Lo Presti S., Mancini M. (2001). Rheology of scleroglucan dispersions. J. Food Eng..

[14] Zhang S., Sun Y., Nie Q., Hu J., Su W., Guo Z., Zhang Y., Nie S. (2023). In Vitro Assessment of the Effect of Four Polysaccharides on Intestinal Bacteria of Mice with Colitis. Food Front..

[15] Kycia K., Chlebowska-Śmigiel A., Szydłowska A., Sokół E., Ziarno M., Gniewosz M. (2020). Pullulan as a Potential Enhancer of Lactobacillus and Bifidobacterium Viability in Synbiotic Low Fat Yoghurt and Its Sensory Quality. LWT.

[16] Ivashchenko O., Khonkiv M., Stabnikov V., Polishchuk G., Marynin A., Buniowska-Olejnik M. (2024). Influence of Starch Products on the Vitality and Activity of Lactic Acid Bacteria in Yogurt. Ukr. Food J..

[17] Tamime A.Y., Robinson R.K. (2007). Tamime and Robinson’s Yoghurt.

[18] Lazaridou A., Serafeimidou A., Biliaderis C.G., Moschakis T., Tzanetakis N. (2014). Structure Development and Acidification Kinetics in Fermented Milk Containing Oat β-Glucan, a Yogurt Culture and a Probiotic Strain. Food Hydrocoll..

[19] Aljewicz M., Majcher M., Nalepa B. (2020). A Comprehensive Study of the Impacts of Oat β-Glucan and Bacterial Curdlan on the Activity of Commercial Starter Culture in Yogurt. Molecules.

[20] Ge Y., Yu X., Zhao X., Liu C., Li T., Mu S., Zhang L., Chen Z., Zhang Z., Song Z. (2024). Fermentation Characteristics and Postacidification of Yogurt by *Streptococcus thermophilus* CICC 6038 and *Lactobacillus delbrueckii* ssp. bulgaricus CICC 6047 at Optimal Inoculum Ratio. J. Dairy Sci..

[21] Brüls M., Foroutanparsa S., Maljaars C.E.P., Olsthoorn M., Tas R.P., Voets I.K. (2024). Investigating the Impact of Exopolysaccharides on Yogurt Network Mechanics and Syneresis through Quantitative Microstructural Analysis. Food Hydrocoll..

[22] Li D., Cui Y., Wu X., Li J., Min F., Zhao T., Zhang J., Zhang J. (2024). *Graduate Student Literature Review*: Network of Flavor Compounds Formation and Influence Factors in Yogurt. J. Dairy Sci..

[23] Liu C., Yang P., Wang H., Song H. (2022). Identification of Odor Compounds and Odor-Active Compounds of Yogurt Using DHS, SPME, SAFE, and SBSE/GC-O-MS. LWT.

[24] Martin N., Berger C., Le Du C., Spinnler H.E. (2001). Aroma Compound Production in Cheese Curd by Coculturing with Selected Yeast and Bacteria. J. Dairy Sci..

[25] Li Y., Zhang L., Wang W. (2012). Formation of Aldehyde and Ketone Compounds during Production and Storage of Milk Powder. Molecules.

[26] Landaud S., Helinck S., Bonnarme P. (2008). Formation of Volatile Sulfur Compounds and Metabolism of Methionine and Other Sulfur Compounds in Fermented Food. Appl. Microbiol. Biotechnol..

[27] Chen C., Zhao S., Hao G., Yu H., Tian H., Zhao G. (2017). Role of Lactic Acid Bacteria on the Yogurt Flavour: A Review. Int. J. Food Prop..

[28] Cheng H. (2010). Volatile Flavor Compounds in Yogurt: A Review. Crit. Rev. Food Sci. Nutr..

[29] Smid E.J., Kleerebezem M. (2014). Production of Aroma Compounds in Lactic Fermentations. Annu. Rev. Food Sci. Technol..

[30] Irigoyen A., Arana I., Castiella M., Torre P., Ibanez F.C. (2005). Microbiological, physicochemical, and sensory characteristics of kefir during storage. Food Chem..

[31] Yao D., Ranadheera C.S., Shen C., Wei W., Cheong L.-Z. (2024). Milk Fat Globule Membrane: Composition, Production and Its Potential as Encapsulant for Bioactives and Probiotics. Crit. Rev. Food Sci. Nutr..

[32] Mykhalevych A., Polishchuk G., Nassar K., Osmak T., Buniowska-Olejnik M. (2022). β-Glucan as a Techno-Functional Ingredient in Dairy and Milk-Based Products—A Review. Molecules.

[33] Cao H., Li R., Shi M., Song H., Li S., Guan X. (2024). Promising Effects of β-Glucans on Gelation in Protein-Based Products: A Review. Int. J. Biol. Macromol..

[34] Geng X., Zhao N., Song X., Wu J., Zhu Q., Wu T., Chen H., Zhang M. (2022). Fabrication and Characterization of Konjac Glucomannan/Oat β-Glucan Composite Hydrogel: Microstructure, Physicochemical Properties and Gelation Mechanism Studies. Molecules.

[35] Hassan A.N., Ipsen R., Janzen T., Qvist K.B. (2003). Microstructure and Rheology of Yogurt Made with Cultures Differing Only in Their Ability to Produce Exopolysaccharides. J. Dairy Sci..

[36] Zhao Y., Fu R., Li J. (2020). Effects of the β-Glucan, Curdlan, on the Fermentation Performance, Microstructure, Rheological and Textural Properties of Set Yogurt. LWT.

[37] Qu X., Nazarenko Y., Yang W., Nie Y., Zhang Y., Li B. (2021). Effect of Oat β-Glucan on the Rheological Characteristics and Microstructure of Set-Type Yogurt. Molecules.

[38] Florczuk A., Dąbrowska A., Aljewicz M. (2022). An Evaluation of the Effect of Curdlan and Scleroglucan on the Functional Properties of Low-Fat Processed Cheese Spreads. LWT.

[39] Macit E., Bakirci I. (2017). Effect of Different Stablizers on Quality Characteristics of the Set-Type Yogurt. Afr. J. Biotechnol..

[40] Aljewicz M., Keklik M., Recio I., Martínez-Sanz M. (2024). Effect of Polysaccharide-Protein Interactions on the Multi-Scale Structure of Hybrid Micellar Casein-Xanthan Gum Systems. Food Hydrocoll..

[41] ISO 13299:2016. https://www.iso.org/standard/58042.html.

[42] ISO 8586:2012. https://www.iso.org/standard/45352.html.

